# Supplementary material for: Solid cyclooctatetraene-based triplet quencher demonstrating excellent suppression of singlet–triplet annihilation in optical and electrical excitation
Source: Nat Commun. 2020 Nov 6;11:5623. doi: 10.1038/s41467-020-19443-z (PMC7648636; doi:10.1038/s41467-020-19443-z)
Supplement: Supplementary file 1 — Supplementary Information [file 41467_2020_19443_MOESM1_ESM.pdf]

Supplementary Information:

Solid Cyclooctatetraene-Based Triplet Quencher Demonstrating Excellent Suppression of Singlet-Triplet Annihilation in Optical and Electrical Excitation

Van T. N. Mai,[1,2,#] Viqar Ahmad,[1,3,#] Masashi Mamada,[4,5,6] Toshiya Fukunaga,[4,5] Atul Shukla,[1,3] Jan Sobus,[1,3] Gowri Krishnan,[7] Evan G. Moore,[2] Gunther Andersson,[7] Chihaya Adachi,[4,5,8,] Ebinazar B. Namdas,[1,3,*] Shih-Chun Lo[1,2,*]*

[1]Centre for Organic Photonics & Electronics, The University of Queensland, Brisbane, QLD 4072, Australia

[2]School of Chemistry and Molecular Biosciences, The University of Queensland, Brisbane, QLD 4072, Australia

[3]School of Mathematics and Physics, The University of Queensland, Brisbane, QLD 4072, Australia

[4]Center for Organic Photonics and Electronics Research (OPERA), Kyushu University, Fukuoka 819-0395, Japan

[5]JST, ERATO, Adachi Molecular Exciton Engineering Project c/o Centre for Organic Photonics and Electronics Research (OPERA), Kyushu University, Nishi, Fukuoka 819-0395, Japan

[6]Academia-Industry Molecular Systems for Devices Research and Education Centre (AIMS), Kyushu University, Nishi, Fukuoka 819-0395, Japan

[7]Flinders Institute for Nanoscale Science and Technology, Flinders University Sturt Road, Bedford Park, Adelaide, SA 5042, Australia

[8]International Institute for Carbon Neutral Energy Research (WPI-I2CNER), Kyushu University, Nishi, Fukuoka 819-0395, Japan

[#]The two authors contributed equally

*Correspondence to: Shih-Chun Lo (s.lo@uq.edu.au),

Ebinazar B. Namdas (e.namdas@uq.edu.au),

Chihaya Adachi (adachi@opera.kyushu-u.ac.jp)

Keywords: organic laser, triplet management, cyclooctatetraene, singlet-triplet annihilation, OLEDs

Table of Contents

Supplementary Section 1. Material Synthesis. S3

Supplementary Section 2. Cyclic Voltametry (CV) details........ S7

Supplementary Section 3. Ultraviolet Photoelectron Spectroscopy details (UPS)........S8

Supplementary Fig. 1. Thermogravimetric analysis thermograms of **mCP-COT**. S9

Supplementary Fig. 2. DSC traces of **mCP-COT** S10

Supplementary Fig. 3. Cyclic voltammograms of **mCP-COT** S11

Supplementary Fig. 4. Ionisation potential and electron affinity of **mCP-COT** from ultraviolet photoelectron spectroscopy (UPS) and inversed photoelectron spectroscopy (IPES) S12

Supplementary Fig. 5. TCSPC PL decay curves for **mCP** and **mCP-COT** in toluene S13

Supplementary Fig. 6. Transient absorption for **mCP** and **mCP-COT**, and comparison of triplet excited-state absorption decay S14

Supplementary Fig. 7. The optimised structures in the ground and excited states, and the excited state energy diagram for COT, **mCP-COT** and mCP. S15

Supplementary Fig. 8. Absorption and PL spectra of **BSBCz-EH** with **mCP-COT**.. .... S16

Supplementary Fig. 9. Neat and blend-film PLQYs of **CBP**, **BSBCz-EH** and **BSBCz-CN-EH** with various **mCP-COT** doping concentrations S17

Supplementary Fig. 10. Relative drop in STA as a function of **mCP-COT** and **ADN** concentrations for **BSBCz-EH** and Alq_3/DCM2, respectively.. .... S18

Supplementary Fig. 11. TCSPC PL decay for **BSBCz-EH** neat and blend (**mCP-COT**) thin films S19

Supplementary Fig. 12. Summary of ASE thresholds with varying concentrations of **mCP-COT**. S20

Supplementary Fig. 13. ASE thresholds of neat and blend films. S22

Supplementary Fig. 14. Simulated singlet density evolution compared with experimental data S23

Supplementary Fig. 15. Comparison of neat and blend OLED electrical characteristics.... S24

Supplementary Table 1. Photophysical properties of **mCP-COT** compared with those of mCP. S25

Supplementary Table 2. Summary of photophysical parameters for **mCP** and **mCP-COT** in toluene S26

Supplementary Table 3. Calculated ground-state and vertical excitation energies (VEE) for **COT**, **mCP-COT** and **mCP** S27

Supplementary Table 4. Calculated vertical excited states for **COT**, **mCP-COT** and **mCP** S28

Supplementary Table 5. Calculated adiabatic excitation energies (AEE) for the singlet and triplet of **COT**, **mCP**, and for the triplet excited states of **COT**, **mCP-COT** and **mCP** S29

Supplementary Table 6. Summary of photophysical parameters for BSBCz-EH neat and blend (mCP-COT) thin films S30

Supplementary Section 4. ^{1}H and ^{13}C NMR spectra of all compounds S31

References. S37

Supplementary Section 1: Material syntheses:

Petroleum with boiling points of 40–60 °C and dichloromethane were distilled prior to use for column chromatography, using Merck LC60A 40–30 silica gel. Solvent ratio used for column chromatography is reported by volume. All ^{1}H spectra were recorded using Bruker Avance 300 or 500 MHz spectrometers in $CDCl_3$. All chemical shifts (δ) were reported in parts per million (ppm) and referenced to the residual solvent peak at δ 7.26 ppm in ^{1}H NMR, and 77.0 ppm for $CDCl_3$ in ^{13}C NMR. Multiplicities were reported as singlet (s), doublet (d), triplet (t), multiplet (m), doublet of doublets (dd) and doublet of triplets (dt); COT-H = cyclooctatetraenyl H, Cz-H = carbazolyl H, Ph-H = phenyl H and V-H = vinyl H. All coupling constants (*J*) were quoted in Hertz (Hz) and rounded to the nearest 0.5 Hz. Melting point (m.p.) was measured in a glass capillary on a BÜCHI Melting Point B-545 and was uncorrected. Infrared spectra were recorded on a Perkin Elmer Spectrum 1000 FT-IR spectrometer with ATR attachment as solid state. Mass spectra were recorded on a Bruker Esquire HCT (High Capacity 3D ion trap) electrospray ionization (ESI) MS or a BRUKER MicrOTof-Q for the accurate mass in ESI mode. Absorption spectra were recorded on a Varian Cary 5000 UV-Vis-NIR spectrophotometer in 10 × 10 mm quartz cuvettes and λ_{abs} values are quoted in nm with shoulders denoted as "sh". Electrochemical studies were conducted using an Epsilon C3 BAS electrochemistry station using glassy carbon working, 0.01 M $AgNO_3$ in acetonitrile reference, and platinum counter electrodes. All measurements were conducted at room temperature with the sample concentration of 1 mM in dichloromethane (distilled from calcium hydride) and 0.1 M tetra-*n*-butylammonium perchlorate as the electrolyte. The solution was deoxygenated with argon and a ferricenium/ferrocene (Fc^{+}/Fc) couple was used as standard. The scan rate was 100 mV s^{-1}. The thermal gravimetric analysis (TGA) measurement was performed on a Perkin Elmer STA 6000 under 20 °C min^{-1} under an inert atmosphere, the TGA temperature was quoted at 5% weight loss as the decomposition temperature. Differential scanning calorimetry (DSC) was used to investigate thermal properties with heating and cooling rates of 200 °C min^{-1} under a nitrogen atmosphere.

9,9'-(5-Bromo-1,3-phenylene)bis(9*H*-carbazole), 1

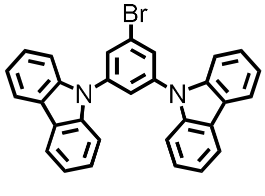

A mixture of 9*H*-carbazole (1.64 g, 9.84 mmol) and potassium *tert*-butoxide (1.30 g, 11.6 mmol) was deoxygenated using vacuum and back-filled with $Ar_{(g)}$. This was repeated three times. Anhydrous DMSO (stirred overnight with 3wt% CaH_2, distilled and stored in 4 Å molecular sieves, 5.0 mL) was added to the mixture and the solution was stirred in an oil bath heated at 120 °C under $Ar_{(g)}$ for 30 minutes, during which the yellow solution turned into orange.

1-Bromo-3,5-difluorobenzene (0.60 mL, 5.21 mmol) was added to the mixture and the reaction was stirred in an oil bath heated at 140 °C under $Ar_{(g)}$ for 2 hours, during which the solution turned into grey with white precipitations. The reaction was cooled to room temperature. Dichloromethane (70 mL) and water (100 mL) were added and the two layers were separated. The aqueous layer was extracted with dichloromethane (2 × 30 mL). All organic layers were combined, washed with water (2 × 150 mL), brine (150 mL), dried over anhydrous magnesium sulfate and filtered. The filtrate was collected and the solvent removed under reduced pressure to reveal an off-white solid. The solid was purified by column chromatography over silica using dichloromethane/light petroleum (1:8) as eluent to give **1** as a white solid (1.76 g, 73%). ^{1}H NMR (500 MHz, $CDCl_3$) δ 7.31–7.35 (4H, m, Cz-H), 7.44–7.48 (4H, m, Cz-H), 7.55 (4H, d, *J* = 8.0, Cz-H), 7.80 (1H, t, *J* = 2.0, Ph-H), 7.87 (2H, d, *J* = 2.0, Ph-H), 8.15 (4H, d, *J* = 7.5, Cz-H); ^{13}C NMR (125 MHz, $CDCl_3$) δ 109.6, 120.6, 120.8, 123.8, 123.9, 124.1, 126.4, 128.7, 140.3, 140.5. These NMR spectra are identical to literature[1,2].

9,9'-(5-(Hex-5-en-1-yl)-1,3-phenylene)bis(9*H*-carbazole), 3

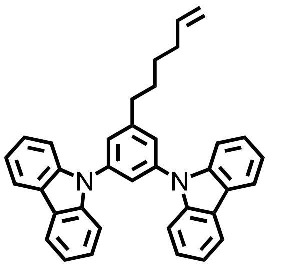

9-Borabicyclo[3.3.1]nonane (0.5 M in THF, 20 mL, 10.0 mmol) was added dropwise to a solution of 1,5-hexadiene (12 mL, 98.7 mmol) under $Ar_{(g)}$ at room temperature. The mixture was stirred at room temperature for 2.5 hours. The excess 1,5-hexadiene and solvent were removed under reduced pressure to give a cloudy slightly viscous liquid. A mixture of the borane from above, **1** (1.45 g, 2.98 mmol), K_2CO_3 (2.05 g, 14.8 mmol) and $Pd(dppf)Cl_2 \cdot CH_2Cl_2$ (93.0 mg, 127 □mol) was deoxygenated using vacuum and back-filled with $Ar_{(g)}$. This was repeated three times. Anhydrous DMF (14 mL) was added to the mixture and the solution was stirred in an oil bath heated at 60 °C under $Ar_{(g)}$ for 16 hours. The reaction was cooled to room temperature. Diethyl ether (80 mL) and water (150 mL) were added to the mixture. The two layers were separated. The aqueous layer was extracted with diethyl ether (2 × 80 mL). All organic layers were combined, washed with water (2 × 150 mL), brine (120 mL), dried over anhydrous magnesium sulfate and filtered. The filtrate was collected and the solvent removed under reduced pressure to reveal a brown-orange gum. The crude was purified by column chromatography over silica using dichloromethane/light petroleum (1:7) as eluent to give **3** as a white solid (1.11 g, 76%); m.p.: 114.5–116.0 °C; ν_{max}(solid)/cm^{-1}: 706, 747, 1227, 1334, 1443, 1462, 1592, 2849, 2931, 3054; λ_{max} (dichloromethane)/nm: 242 (logε / dm^{3} mol^{-1} cm^{-1} 4.98), 260 sh (4.54), 283 sh (4.57), 293 (4.43), 327 (3.96), 340 (4.03). ^{1}H NMR (500 MHz, $CDCl_3$) δ 1.59–1.65 (2H, m, CH_2), 1.83–1.90 (2H, m, CH_2), 2.18–2.23 (2H, m, CH_2), 2.90 (2H, t, *J* = 7.5, Ph-CH_2), 5.03–5.13 (2H, m,

V-H), 5.86–5.94 (1H, m, V-H), 7.34–7.38 (4H, m, Cz-H), 7.48–7.52 (4H, m, Cz-H), 7.57 (2H, d, J = 2.0, Ph-H), 7.61 (4H, d, J = 8.5, Cz-H), 7.71 (1H, t, J = 2.0, Ph-H), 8.21 (4H, d, J = 8.0, Cz-H); ^{13}C NMR (125 MHz, $CDCl_3$) δ 28.4, 30.6, 33.5, 35.7, 109.7, 114.7, 120.2, 120.4, 122.5, 123.5, 125.8, 126.1, 138.5, 139.1, 140.6, 146.4; m/z (ESI): calculated for $C_{36}H_{30}N_2$ [M]: 490.2 (100%), 491.2 (39%), 492.3 (7%); found $C_{36}H_{30}N_2$ [M]: 490.7 (100%), 491.6 (53%), 492.6 (20%).

(1E,3Z,5Z,7Z)-1-Bromocycloocta-1,3,5,7-tetraene, 5[3]

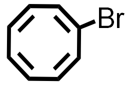

A solution of cyclooctatetraene (8.4 mL, 74.6 mmol) in anhydrous dichloromethane (50 mL) was cooled to –70 °C under argon. A solution of bromine (3.8 mL, 74.2 mmol) in anhydrous dichloromethane (40 mL) was slowly added over 10 minutes into the cold solution of cyclooctatetraene. The solution was stirred at –70 °C under argon for 1 hour to give a pale-yellow solution. A suspension of potassium *tert*-butoxide (11.2 g, 100 mmol) in anhydrous tetrahydrofurane (40 mL) was added to the cold yellow solution of cyclooctatetraene. The mixture was then stirred at –60 °C under argon for 3 hours and poured into iced water (200 mL). The mixture was extracted with diethyl ether (3 × 250 mL), washed with water (3 × 400 mL), dried over anhydrous magnesium sulfate and filtered. The filtrate was collected and the solvent removed under reduced pressure to reveal an amber yellowish oil. ^{1}H NMR suggested that the crude mixture contained both the desired product (≈80%) and starting material cyclooctatetraene (≈20%). The mixture was used for next step without further purification.

9,9'-(5-(6-((1Z,3Z,5Z,7Z)-Cycloocta-1,3,5,7-tetraen-1-yl)hexyl)-1,3-phenylene)bis(9*H*-carbazole), mCP-COT

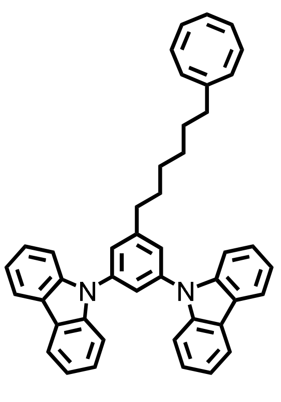

A solution of **3** (803 mg, 1.64 mmol) in anhydrous tetrahydrofuran (2.5 mL) was cooled to 0–2 °C under argon. 9-Borabicyclo[3.3.1]nonane (0.5 M in tetrahydrofuran, 5.0 mL, 2.50 mmol) was added dropwise to the cold solution. The resulting light yellow mixture was stirred at room temperature for 3.5 hours. DMF/H_2O (5.0 mL, 4:1 v/v, bubbled with nitrogen gas for 3 h), **5** (0.7 mL, 5.43 mmol), K_2CO_3 (894 mg, 6.65 mmol) and Pd(dppf)Cl_2·CH_2Cl_2 (72.1 mg, 100 μmol) were added to the mixture. The reaction was then deoxygenated using vacuum and back-filled with $Ar_{(g)}$. This was repeated three times. The solution was stirred in an oil bath heated at 55 °C under $Ar_{(g)}$ for 17 hours. The resulted brown-reddish solution was cooled to room temperature and volatile solvent was

removed under reduced pressure. The mixture was diluted with ethyl acetate (150 mL) and washed with water (100 mL). The aqueous layer was extracted with ethyl acetate (100 mL). All organic layers were combined, washed with water (5 × 100 mL), brine (50 mL), dried over anhydrous magnesium sulfate and filtered. The filtrate was collected and the solvent removed in vacuo to reveal a viscous brown-yellowish oil. The crude was purified by column chromatography over silica using petroleum as eluent to give **mCP-COT** as a colourless solid (594 mg, 61%); m.p.: 71.9–73.1 °C; T_d(5%) = 408 °C; ν_{max}(solid)/cm^{-1}: 704, 722, 745, 1227, 1311, 1332, 1442, 1461, 1591, 2853, 2926, 2998, 3049; λ_{max} (dichloromethane)/nm: 241 (logε / dm^3 mol^{-1} cm^{-1} 4.93), 289 sh (4.53), 293 (4.60), 327 (3.92), 340 (3.98). ^{1}H NMR (500 MHz, $CDCl_3$) δ 1.40–1.49 (6H, m, CH_2), 1.75–1.82 (2H, m, CH_2), 2.03–2.07 (2H, m, -$\underline{CH_2}$-COT), 2.84 (2H, t, *J* = 8.0, Ph-CH_2), 5.54 (1H, br s, COT-H), 5.70–5.80 (6H, m, COT-H), 7.30–7.34 (4H, m, Cz-H), 7.43–7.47 (4H, m, Cz-H), 7.50–7.56 (6H, m, Ph-H & Cz-H), 7.64 (1H, t, *J* = 2.0, Ph-H), 8.16 (4H, d, *J* = 7.5, Cz-H); ^{13}C NMR (125 MHz, $CDCl_3$) δ 28.3, 28.8, 29.1, 31.2, 35.8, 37.6, 109.7, 120.2, 120.4, 122.4, 123.5, 125.8, 126.07, 126.14, 130.8, 131.1, 131.9, 132.4, 134.4, 139.0, 140.6, 144.5, 146.7; m/z (ESI): calculated for $C_{44}H_{38}N_2$ [M]: 594.3 (100%), 595.3 (48%), 596.3 (11%); found $C_{44}H_{38}N_2$ [M]: 594.4 (100%), 595.4 (53%), 596.4 (28%); $C_{44}H_{38}N_2$ requires C, 88.9; H, 6.4; N, 4.7%; found: C, 88.9; H, 6.4; N, 4.7%.

Supplementary Section 2: Cyclic Voltammetry (CV) experiment details

Electrochemical properties of **mCP-COT** were studied by using cyclic voltammetry (CV). As shown in **Supplementary Fig. 3**, two chemically reversible oxidations at 0.6 and 0.9 V (*versus* ferrocenium/ferrocene couple) were observed in dichloromethane, likely corresponding to the oxidations of the carbazolyl units[4,5]. We were not able to obtain reversible reduction for **mCP-COT** (see the best reduction wave in tetrahydrofuran in **Supplementary Fig. 3**). Hence, differential pulse voltammetry (DPV) was employed to show a reduction at about −2.6 V *versus* ferrocenium/ferrocene couple, which is likely arisen from the COT moiety[6]. However, it should be noted that due to our non-conjugated connection of the two electroactive mCP and COT species here, the 1st redox potentials gap (*i.e.*, ≈3.2 eV) can not be directly correlated to its optical gap (**Fig. 2a**)—see our computational results in **Supplementary Fig. 7**, where the predominated transition in absorption and emission was arisen from mCP unit of **mCP-COT**. This was further supported by the fact that the crossover point energy (*i.e.*, 342 nm ≈3.6 eV) of the absorption and PL spectra of **mCP-COT** in toluene (**Fig. 2a**) is essentially the same as the difference (≈3.6 eV) of the 1st redox potentials of **mCP** from CV measurements[4]. Namely, the emission of **mCP-COT** was originated from the mCP unit, as also supported by the essentially identical solution absorption and emission spectra with **mCP** in **Fig. 2a**.

Supplementary Section 3: Ultraviolet Photoelectron Spectroscopy (UPS) details

Ultraviolet photoelectron spectroscopy (UPS) was employed to determine the valence electron structure[7]. UPS probes the top 2.5–3 nm of the sample, allowing for determining the work-function (WF) of samples and the position of the highest occupied molecular orbital (E_{HOMO}). The WF of the samples was determined from the secondary electron cut-off of the UPS spectra. Deconvolution of the secondary electron cut-off. The secondary electron cut-off was interpolated with a linear function. The WF is determined from the intersection of the linear function with the energy axis. E_{HOMO} was determined at the low binding energy cut-off. Inverse photoemission spectroscopy (IPES) is based on an electron in/photon out mechanism[8,9]. In an IPES experiment, an electron beam is directed onto a sample. Detection of the emitted photons as function of their kinetic energy allows determining the energy of E_{LUMO} of an organic compound. E_{LUMO} was determined as the intersection of base line and linear fitting line of the peak onset.

Hence, the electronic characterisation of mCP-COT neat films was performed by using UPS and IPES. **Supplementary Fig. 4** shows the secondary electron cut-off (SE cut-off) of the UPS spectrum and the HOMO and LUMO onsets from UPS and IPES. The SE cut-off was measured around 17.7±0.1 eV, resulting in a WF of 3.5±0.1 eV. The HOMO onset and thus EHOMO was found at 2.4±0.1 eV. The ionisation potential is given by IP = WF + EHOMO. IP thus is given as 5.9±0.2 eV. From the IPES measurements, the LUMO onset and thus ELUMO was found to be at 0.0±0.1 eV. However, this will only give a small HOMO–LUMO gap (2.4 eV), compared to that (≈3.2 eV) obtained from above CV measurements in solution. Hence, the first onset as well as the low intensity in the IPE spectrum between 0 and -0.9 eV are likely arisen from aggregates of the material in the neat films instead of single **mCP-COT** molecules. Accordingly, we assigned the LUMO as -0.9±0.1 eV (**Supplementary Fig. 4**). This endows a HOMO–LUMO gap of 3.3±0.2 eV. The electron affinity (EA) is given by EA = IP – gap, resulting in an EA of 2.6±0.2 eV.

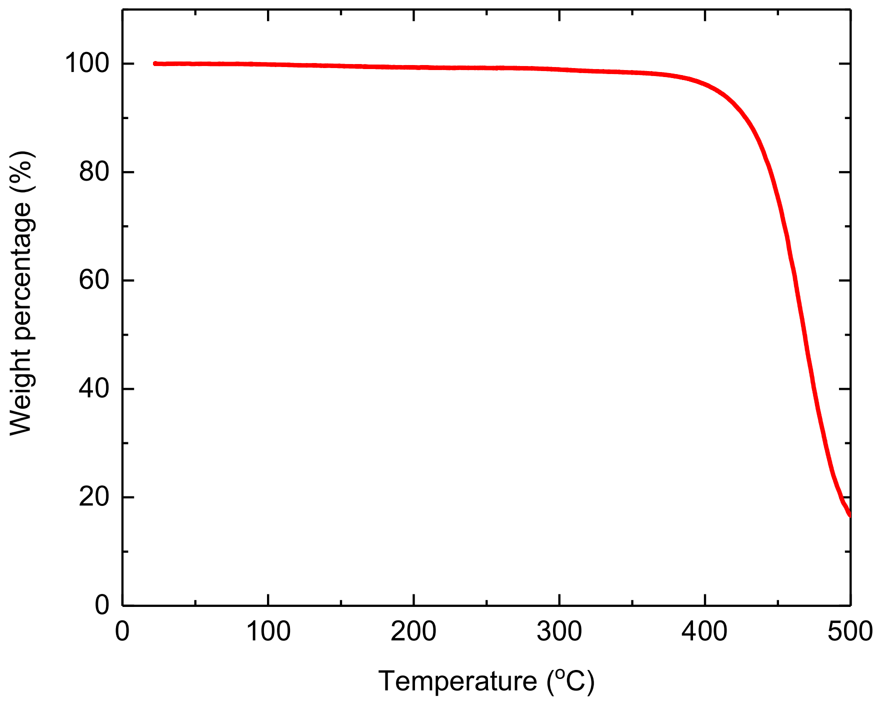

Supplementary Fig. 1: Thermogravimetric analysis (TGA) trace of mCP-COT with a heating rate of 10 °C min^{-1} under nitrogen.

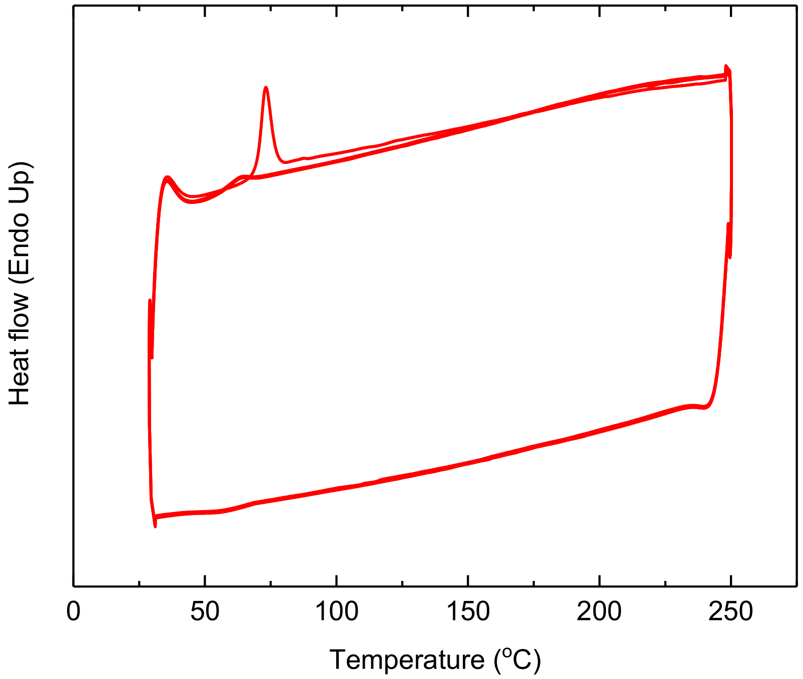

Supplementary Fig. 2: DSC traces of mCP-COT. Scanned from 30 °C to 250 °C with a scan rate of 100 °C min^{-1}. A glass transition temperature (T_g) was observed in the 2nd heating cyle, which is distinguished from that (73 °C) of the melting temperature (an endothermic peak in the 1st heating cycle).

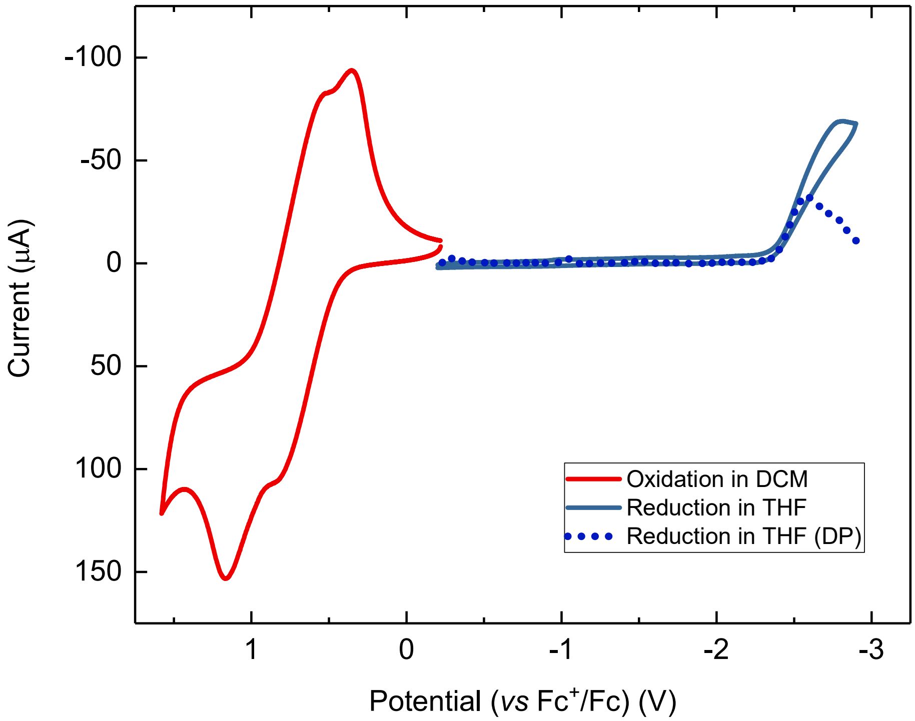

Supplementary Fig. 3: Cyclic voltammograms of mCP-COT. Quoted against ferrocenium/ferrocene (Fc^+/Fc) couple; showing oxidation in dichloromethane (red solid line) and reduction in tetrahydrofuran (blue solid line) (1 mM). Differential pulse (DP) voltammetry was also conducted for reduction of **mCP-COT** in tetrahydrofuran (dotted blue line). Measurements were conducted with 0.1 M tetra-*n*-butylammonium perchlorate electrolyte; working electrode = glassy carbon; reference electrode = 0.01 M $AgNO_3$ in acetonitrile; counter electrode = platinum; scan rate = 100 mV s^{-1}. While the oxidations likely corresponded to the carbazolyl units of **mCP-COT**[4,5], the reduction can be ascribed to the COT moiety[6].

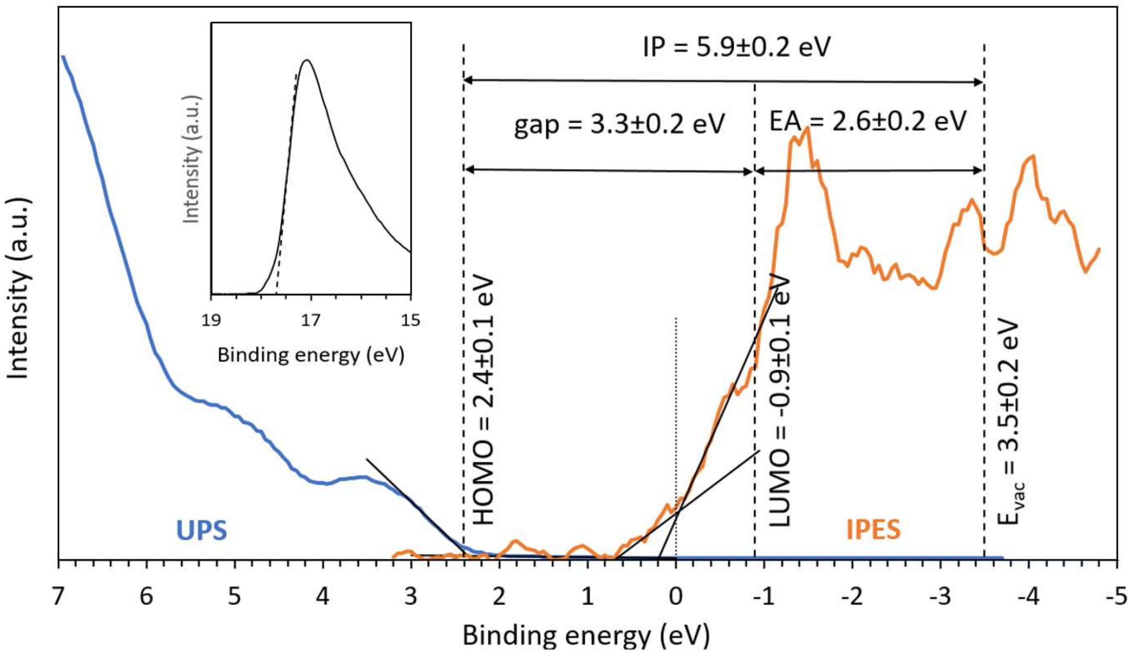

Supplementary Fig. 4: UPS and IPES spectra of mCP-COT. E_{HOMO} was been determined from the UP spectrum as the low binding energy cut-off. E_{LUMO} was determined from the IPE spectrum as the intersection of base line and linear fitting line of the peak onset. The WF of the sample was determined from the secondary electron cut-off of the UP spectrum (see inset). These gave an IP and EA of **mCP-COT** neat films as 5.9±0.2 and 2.6±0.2 eV, respectively.

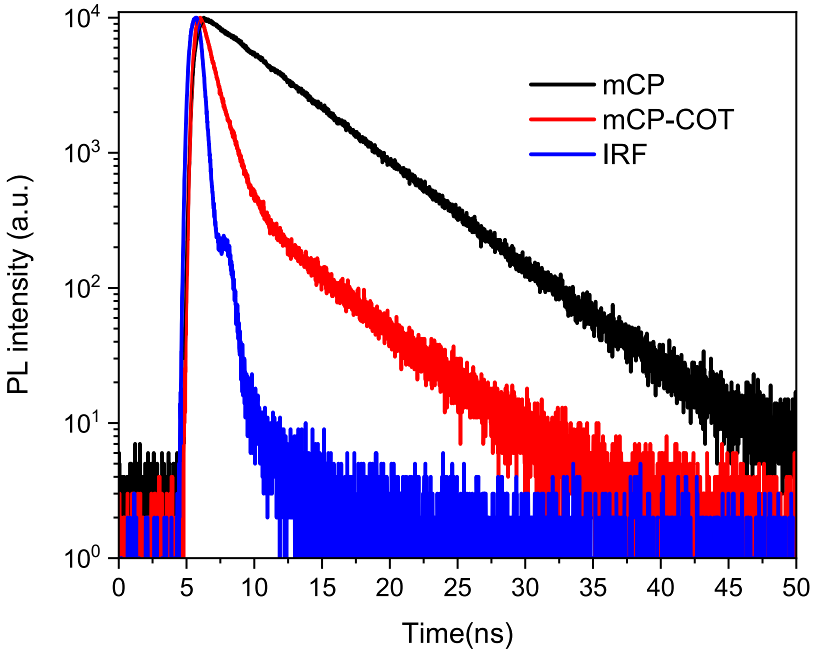

Supplementary Fig. 5: TCSPC PL decay curves for mCP and mCP-COT in toluene.

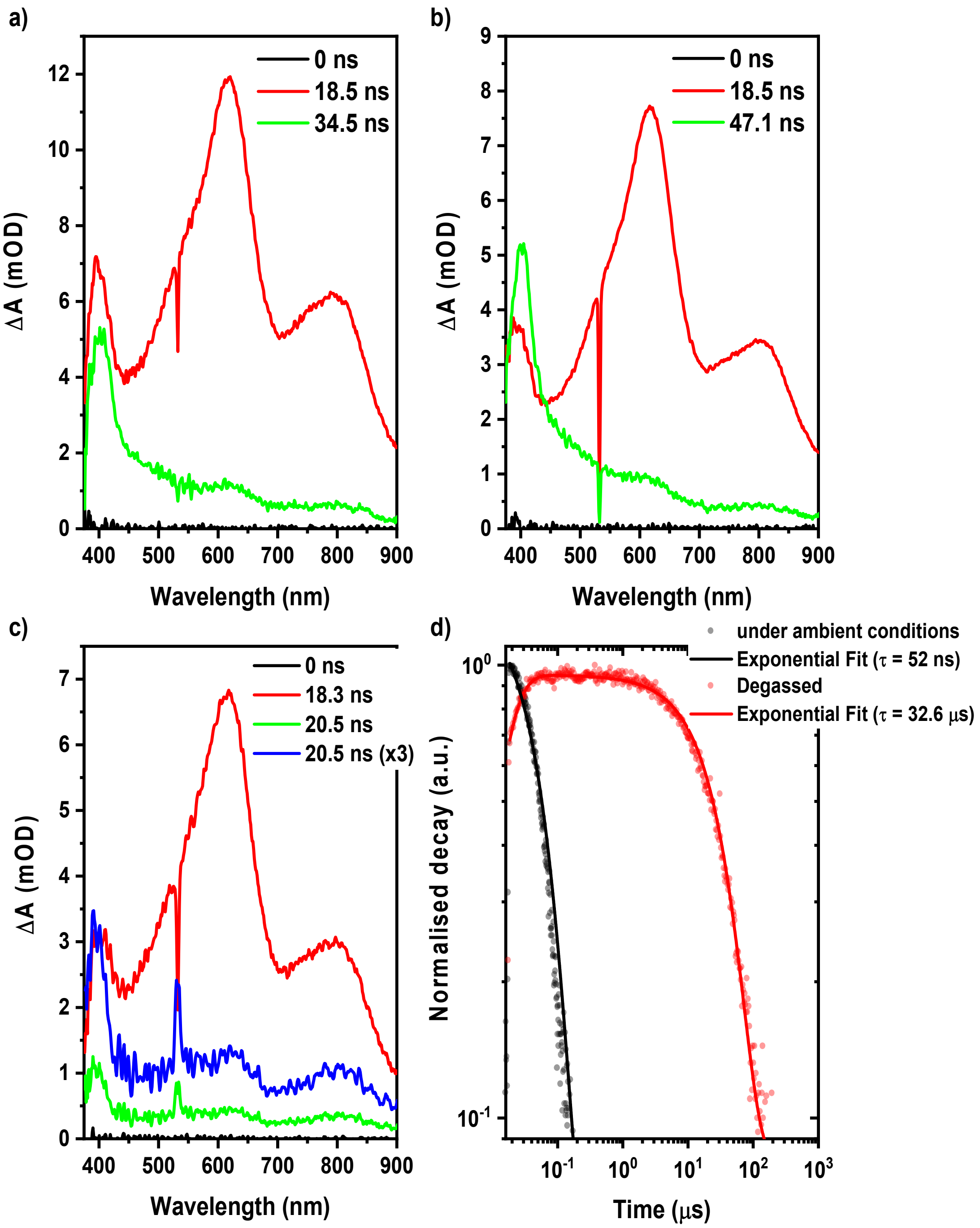

Supplementary Fig. 6: Results of transient absorption spectroscopy. Two distinct transient absorption bands obtained in case of (**a**) **mCP** under ambient conditions (aerated) (**b**) **mCP** under degassed condition (deoxygenated) and (**c**) **mCP-COT** (under degassed conditions). **d** Comparison of triplet excited-state absorption decay (at 400 nm) under ambient and degassed conditions in case of **mCP**.

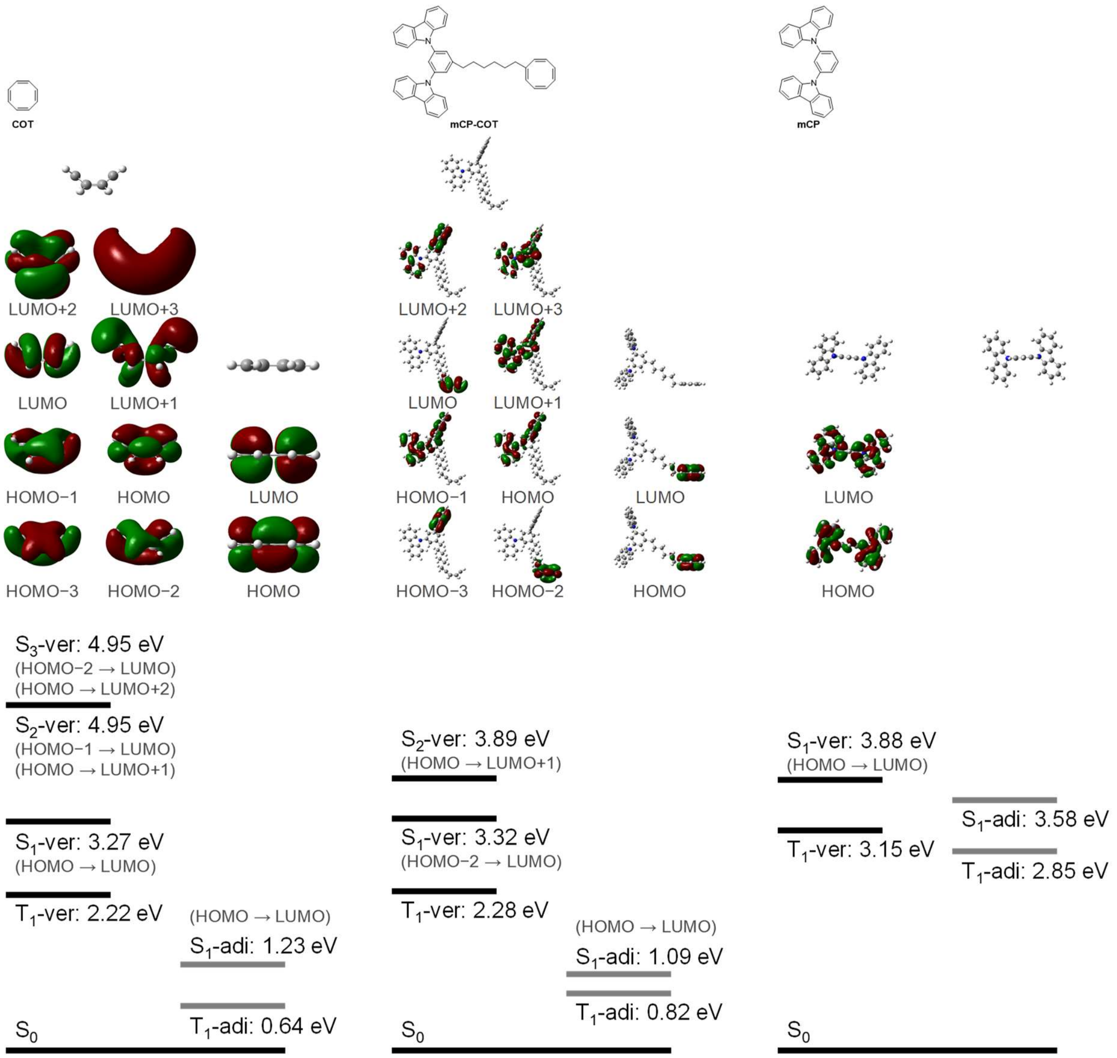

Supplementary Fig. 7: The optimised structures in the ground state (left) and the excited state (right), and the excited state energy diagram for COT, mCP-COT and mCP. Ver- = vertical excitation and adi- = adiabatic excitation.

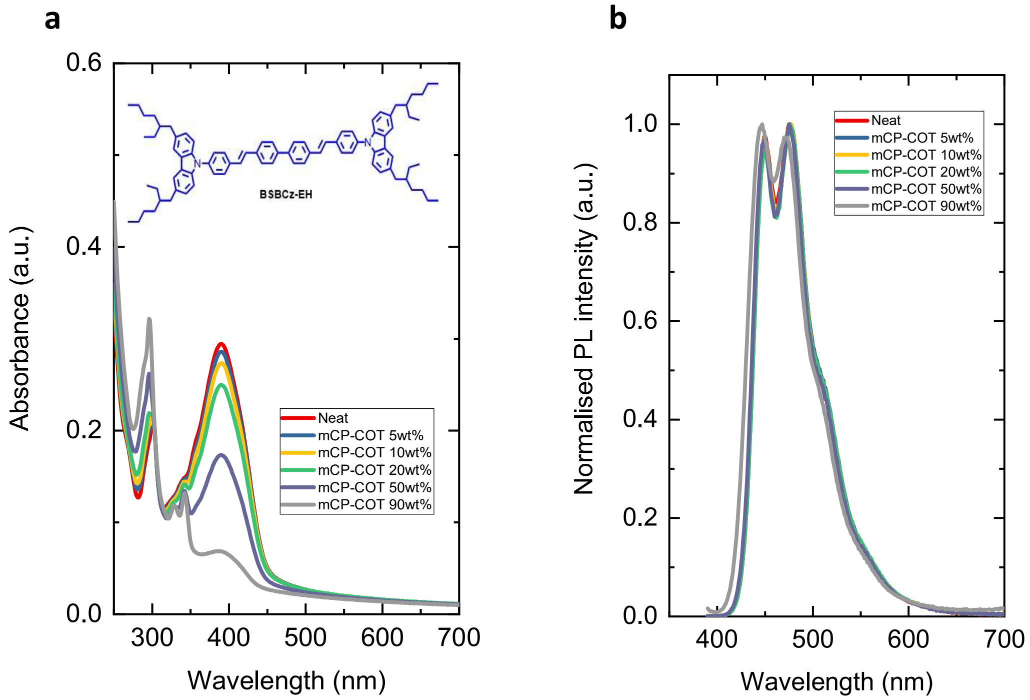

Supplementary Fig. 8: Film absorption and PL spectra of BSBCz-EH for varying concentrations of mCP-COT. **a** Absorption spectrum of **BSBCz-EH** neat and blend films with varying **mCP-COT** doping concentrations (1wt%, 3wt%, 5wt%, 10wt%, 20wt%, 50wt% and 90wt%), inset shows chemical structure of the solution-processable blue-emitting **BSBCz-EH** dye. Excitation wavelength = 380 nm. **b** Normalsised PL intensity (bottom) spectra of **BSBCz-EH** neat and blend films with varying **mCP-COT** doping concentrations (1wt%, 3wt%, 5wt%, 10wt%, 20wt%, 50wt% and 90wt%).

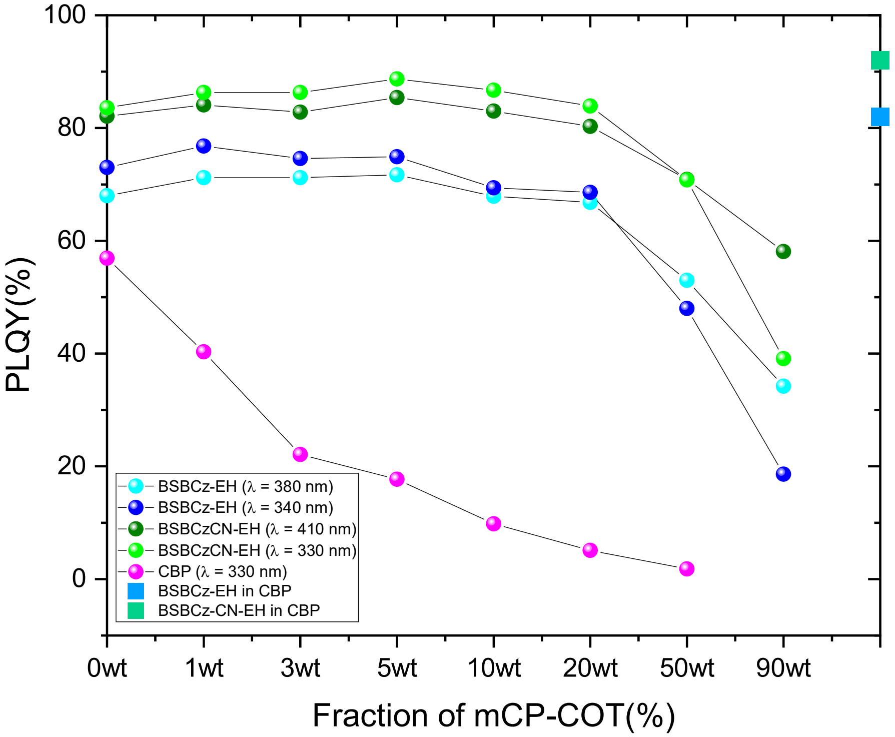

Supplementary Fig. 9: Neat and blend-film PLQYs of CBP, BSBCz-EH and BSBCz-CN-EH with various mCP-COT doping concentrations (*i.e.*, 0%, 1wt%, 3wt%, 5wt%, 10wt%, 20wt%, 50wt% and 90wt%). Excitation wavelength = 330 nm for **CBP**, 380 and 340 nm for **BSBCz-EH**, and 410 and 330 nm for **BSBCz-CN-EH**. The blue and green squares (at top right corner) show blend-film PLQYs of 5wt% **BSBCz-EH** and **BSBCz-CN-EH** in **CBP**, respectively.

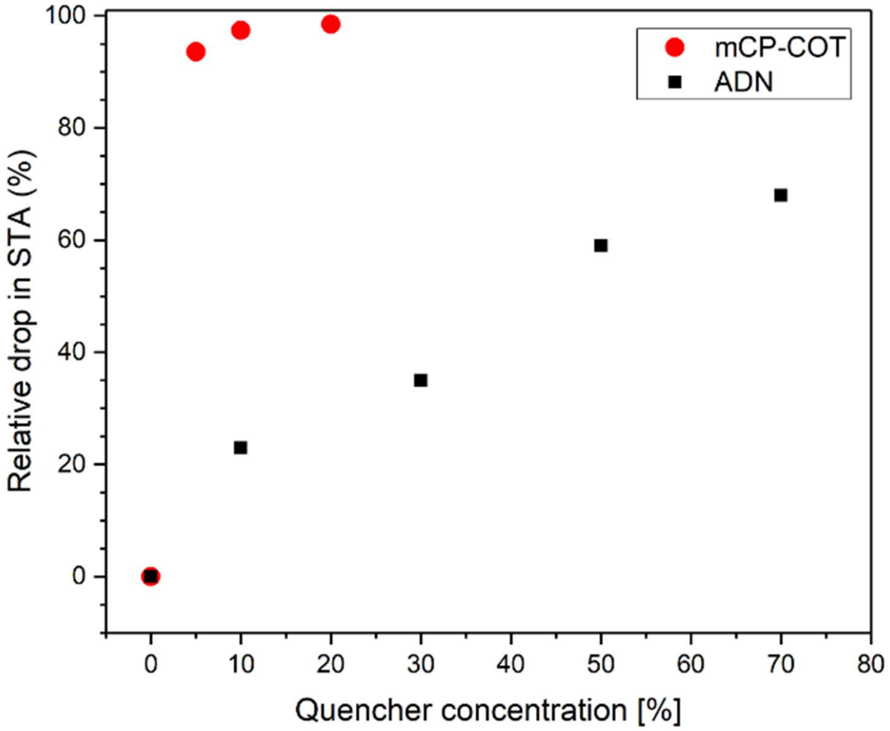

Supplementary Fig. 10: Relative drop in STA as a function of mCP-COT and ADN quencher concentrations for BSBCz-EH and Alq3/DCM2[10], respectively. In the absence of STA, there would be no singlet-triplet interaction between populations; therefore, the singlet population should shortly (under 1 µs) saturate at a steady value where the positive pumping term is balanced out by negative fluorescent ISC and SSA terms (assuming positive contribution of TTA to be negligible) and there is no impact of growing triplet population. Since the singlet population directly correlates to the light intensity, one can treat the difference between peak and steady state in a neat film as a total (*i.e.*, 100%) loss due to STA in a system without triplet quencher. Then, the relative decrease in STA plotted against the quencher concentration can be a rough measure of how successful the triplet manager is in the system.

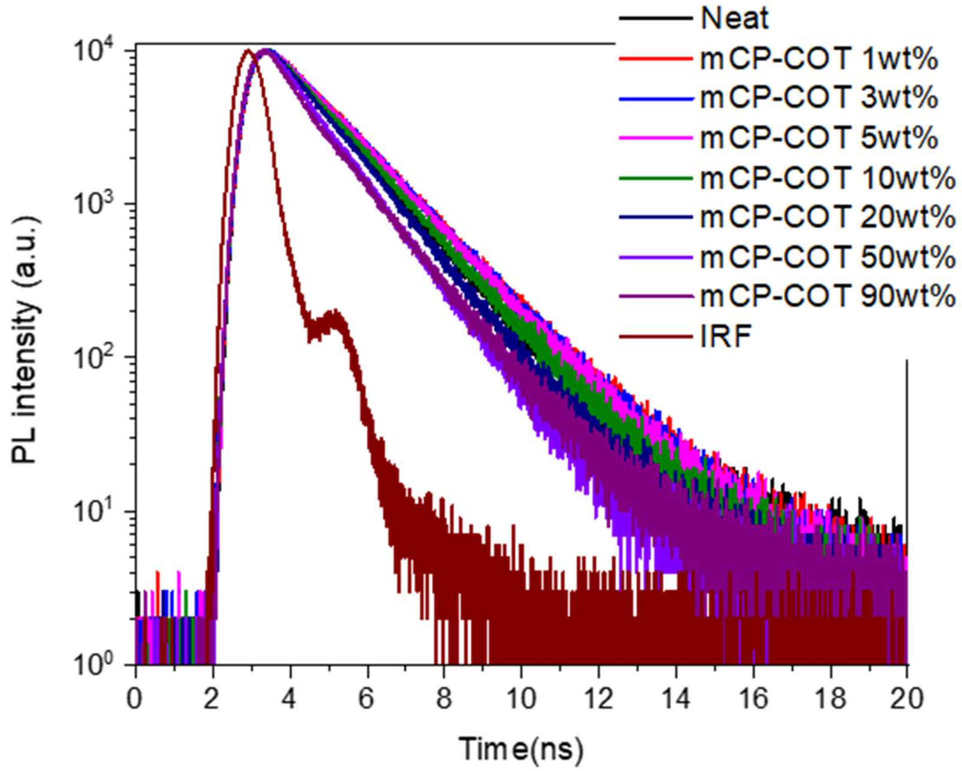

Supplementary Fig. 11: TCSPC PL decay curves for BSBCz-EH neat and blend (mCP-COT) thin films.

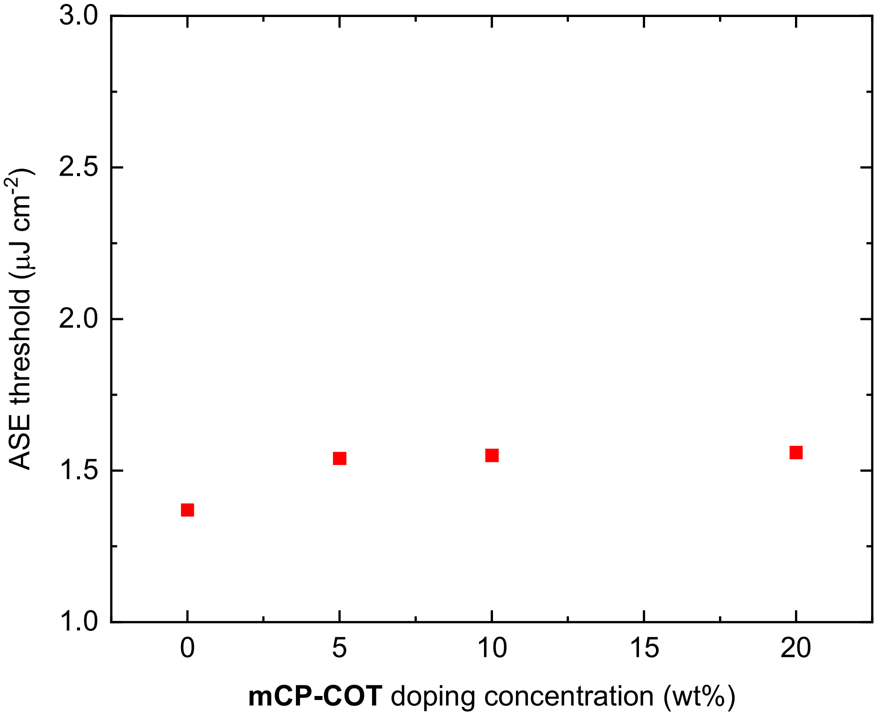

Supplementary Fig. 12: Summary of ASE thresholds with varying concentrations of mCP-COT. Comparable ASE thresholds were achieved in **BSBCz-EH** neat and blend films with **mCP-COT** at 5wt%, 10wt% and 20wt% blend concentrations.

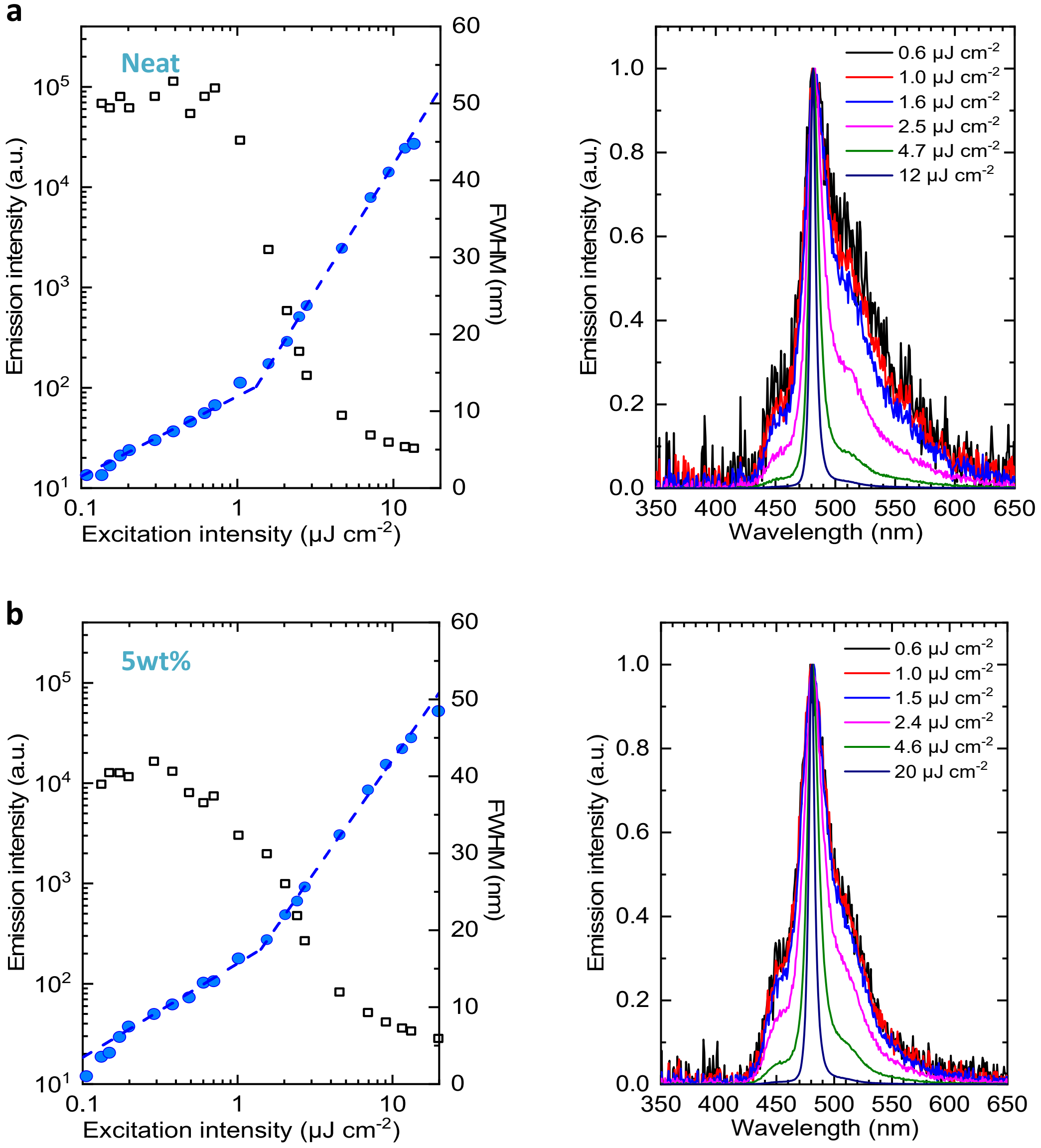
a
Neat
Emission intensity (a.u.)
FWHM (nm)
Excitation intensity (μJ cm^{-2})
0.6 μJ cm^{-2}
1.0 μJ cm^{-2}
1.6 μJ cm^{-2}
2.5 μJ cm^{-2}
4.7 μJ cm^{-2}
12 μJ cm^{-2}
Wavelength (nm)
b
5wt%
0.6 μJ cm^{-2}
1.0 μJ cm^{-2}
1.5 μJ cm^{-2}
2.4 μJ cm^{-2}
4.6 μJ cm^{-2}
20 μJ cm^{-2}

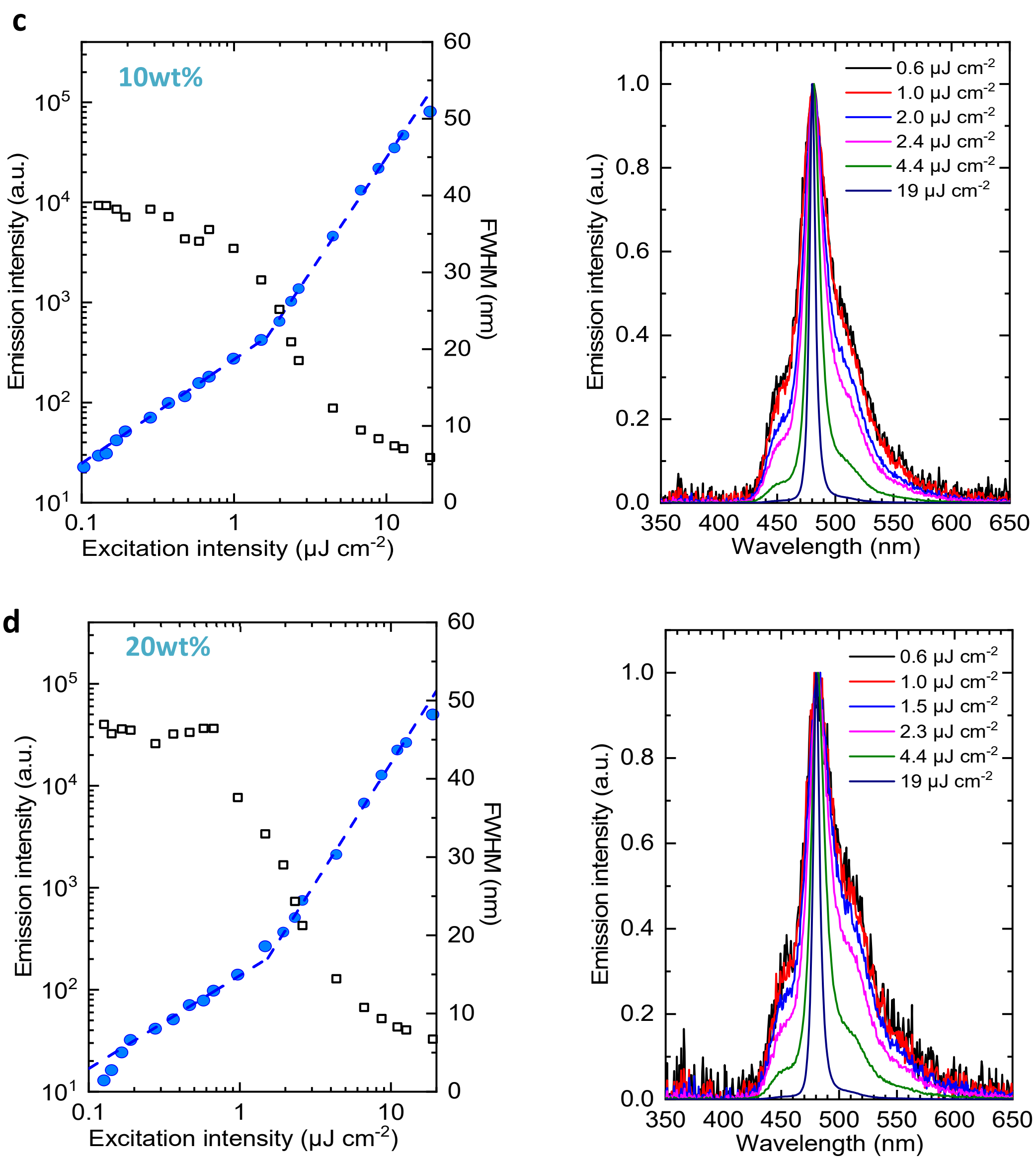

Supplementary Fig. 13: ASE thresholds of neat and blend films. Comparable ASE thresholds were achieved in **BSBCz-EH** (**a**) neat film and blend film with (**b**) 5wt%, (**c**) 10wt% and (**d**) 20wt% of **mCP-COT**. ASE thresholds were estimated from the abrupt change in the slope of input-output intensity (in logarithmic-logarithmic scale) together with significant decrease in full-width at half-maximum (FWHM) (left); photoluminescence spectra at excitation powers below and above ASE threshold showing spectral narrowing with increasing pump intensities (right).

Rate equations:

$$\frac{dn}{dt} = \frac{j(t)}{ed} - k_L n^2$$

$$\frac{dS_1}{dt} = \frac{1}{4}k_L n^2 - k_S S_1 - k_{SS} S_1^2 - k_{ST} S_1 T_1 - 2k_{SP} n S_1 - k_{ISC} S_1 + \frac{1}{4} k_{TT} T_1^2$$

$$\frac{dT_1}{dt} = \frac{3}{4}k_L n^2 - k_T T_1 - \frac{5}{4}k_{TT} T_1^2 - 2k_{TP} n T_1 + k_{ISC} S_1 - k_{mCP\text{-}COT} T_1$$

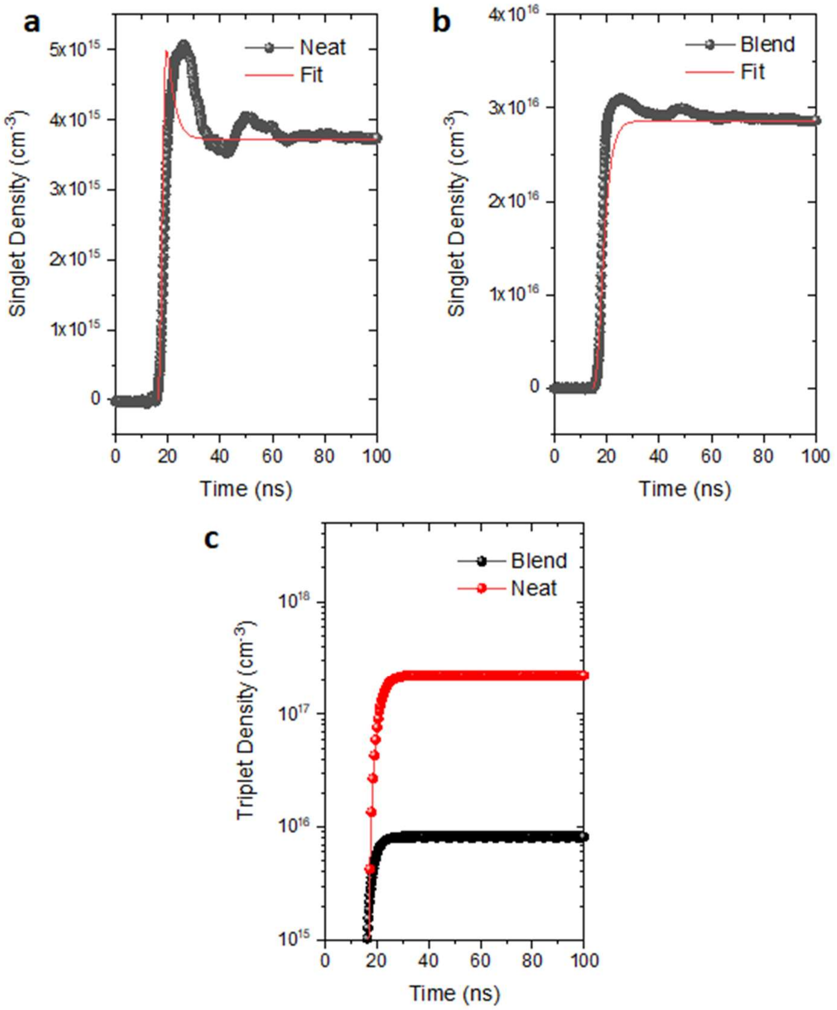

Supplementary Fig. 14: Simulated singlet density evolution over time compared with experimental data at 50 A cm^{-2}. **a** Evolution of singlets in a neat device, actual signal (black) vs simulated fit (red); STA rate recovered from simulation was 4.3×10^{-8} cm^3 s^{-1}. **b** EL fitting for a 2wt% **mCP-COT** blend device actual signal (black) vs simulated fit (red) with the same STA rate (as neat is shown). A new parameter $k_{mCP\text{-}COT}$ was introduced in the blend case signifying triplet depopulation rate due to COT. $k_{mCP\text{-}COT}$ was found to be 1×10^{10} s^{-1}. **c** Evolution of triplet population in neat and blend case is shown depicting efficient triplet recycling in the blend case.

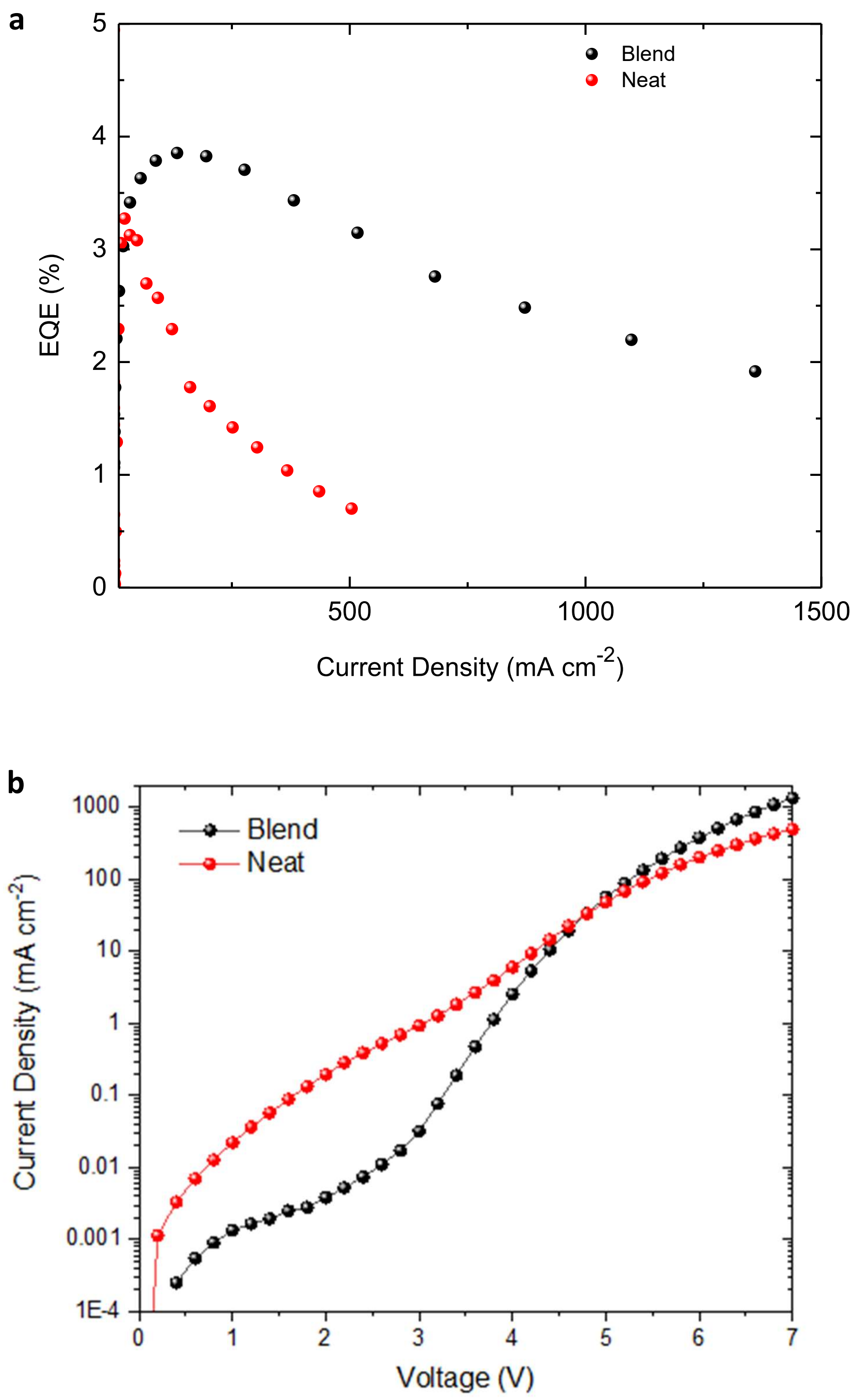

Supplementary Fig. 15: Comparison of neat and blend OLED electrical characteristics. a The graph shows a comparison of neat and 2wt% **mCP-COT** blend OLED EQEs for **BSBCz-EH** where a much sharper roll-off can be seen for neat device (red) as compared to blend (black). Also the blend devices can be seen to sustain higher current densities as compared to the neat device before DC breakdown. **b** Compared neat and blend OLED J-V characteristics, showing similar behaviours in the operating voltage range.

Supplementary Table 1. Photophysical properties of mCP-COT in toluene solution compared with those of mCP.

	λ_{abs} (nm)	λ_{PL} (nm)	PLQY (%)	τ (ns)
**mCP-COT**	293, 327, 340	344, 360, 378	6±3	1.5
**mCP**	294, 325, 339	343, 359, 377	43±3	5.3

Supplementary Table 2. Summary of photophysical parameters for mCP and mCP-COT in toluene.

	**PLQY** (%)	**Lifetime** (ns)	$\boldsymbol{k}_{\mathbf{r}}$ (s^{-1})
**mCP**	43	5.33	8.1×10^7
**mCP-COT**	6	1.46 (0.014, 0.985, 5.67)	4.1×10^7

Supplementary Table 3. Calculated ground-state and vertical excitation energies (VEE) for COT, **mCP-COT** and **mCP** at the B3LYP/6-31+G(d,p) level using polarisable continuum model (PCM).

Compound	PCM	Ground-state S_0 [eV]	S_1-vertical [eV]	Singlet VEE [eV]	T_1-vertical [eV]	Triplet VEE [eV]
**COT**	Gas phase	−8424.942	−8421.669	3.273	−8422.724	2.218
	$CHCl_3$	−8425.006	−8421.761	3.245	−8422.804	2.203
	Cyclohexane	−8424.975	−8421.719	3.256	−8422.765	2.210
	MeOH	−8425.036	−8421.800	3.236	−8422.840	2.196
	THF	−8425.017	−8421.777	3.241	−8422.818	2.199
	Toluene	−8424.982	−8421.730	3.253	−8422.774	2.208
**mCP-COT**	Gas phase	−49230.236	−49226.920	3.316	−49227.952	2.285
	$CHCl_3$	−49230.539	−49227.254	3.285	−49228.272	2.267
	Cyclohexane	−49230.421	−49227.122	3.298	−49228.145	2.276
	MeOH	−49230.596	−49227.321	3.275	−49228.337	2.259
	THF	−49230.527	−49227.246	3.282	−49228.263	2.264
	Toluene	−49230.447	−49227.152	3.295	−49228.173	2.274
**mCP**	Gas phase	−34418.696	−34414.812	3.884	−34415.546	3.150

Supplementary Table 4. Calculated vertical excited states for COT, **mCP-COT** and **mCP** at the B3LYP/6-31+G(d,p) level. [a] H = HOMO, L = LUMO.

Compound	Excited State	VEE [eV]	Wavelength [nm]	*f*	Composition (%)[a]
**COT**	S1	3.2730	378.80	0.0000	H → L (100)
	S2	4.9545	250.25	0.0002	H−1 → L (52), H → L+1 (44)
	S3	4.9545	250.25	0.0002	H−2 → L (52), H → L+2 (44)
	S4	5.2216	237.44	0.0000	H → L+3 (99)
	S5	5.6002	221.39	0.0959	H → L+1 (27), H → L+5 (24), H−2 → L (14), H → L+2 (12)
	S6	5.6002	221.39	0.0959	H → L+2 (27), H → L+4 (24), H−1 → L (14), H → L+1 (12)
	T1	2.2180	559.00	0.0000	H → L (95)
	T2	3.5493	349.32	0.0000	H−1 → L (63), H → L+1 (22)
	T3	3.5493	349.32	0.0000	H−2 → L (63), H → L+2 (22)
	T4	4.6036	269.32	0.0000	H−3 → L (50)
	T5	4.8960	253.24	0.0000	H−1 → L (32), H → L+1 (62)
	T6	4.8960	253.24	0.0000	H−2 → L (32), H → L+2 (62)
**mCP-COT**	S1	3.3162	373.87	0.0021	H−2 → L (100)
	S2	3.8944	317.37	0.0298	H → L+1 (57), H → L+3 (28)
	S3	3.9031	317.65	0.0001	H−1 → L (46), H → L (54)
	S4	3.9174	316.50	0.0001	H−1 → L+1 (47), H → L+2 (33)
	S5	3.9214	316.17	0.0000	H−1 → L (53), H → L (45)
	S6	3.9447	314.30	0.1362	H−1 → L+2 (34), H → L+3 (48)
	T1	2.2846	542.71	0.0000	H−2 → L (94)
	T2	3.1501	393.58	0.0000	H−4 → L+1 (38), H−4 → L+2 (18), H−4 → L+3 (18)
	T3	3.1505	393.54	0.0000	H−3 → L+1 (11), H−3 → L+2 (54)
	T4	3.2745	378.64	0.0000	H−1 → L+1 (47), H → L+2 (31)
	T5	3.2888	376.99	0.0000	H−1 → L+2 (43), H → L+1 (33)
	T6	3.3765	367.20	0.0000	H−1 → L+3 (45)
**mCP**	S1	3.8843	319.19	0.0775	H → L (69), H → L+2 (28)
	S2	3.9150	316.69	0.0008	H−1 → L (58), H → L+1 (31)
	S3	3.9509	313.81	0.0631	H−1 → L+1 (49), H → L+2 (36)
	S4	3.9722	312.13	0.1560	H−1 → L+3 (91)
	S5	3.9986	310.07	0.0483	H−1 → L+2 (58), H → L+1 (18), H → L+3 (16)
	S6	4.0109	309.12	0.0020	H → L+3 (71), H−1 → L (18)
	T1	3.1499	393.61	0.0000	H−3 → L (15), H−3 → L+2 (22), H−2 → L+1 (34)
	T2	3.1499	393.61	0.0000	H−3 → L+1 (34), H−2 → L (15), H−2 → L+2 (22)
	T3	3.2788	378.14	0.0000	H−1 → L (48), H → L+1 (35)
	T4	3.3011	375.58	0.0000	H−1 → L+1 (44), H → L (33), H → L+2 (14)
	T5	3.3687	368.05	0.0000	H−1 → L+2 (48), H → L+1 (12), H → L+3 (13)
	T6	3.6798	336.93	0.0000	H−1 → L+3 (38), H → L (15), H → L+2 (36)

Supplementary Table 5. Calculated adiabatic excitation energies (AEE) at the B3LYP/6-31+G(d,p)//CIS/6-31+G(d,p) for the singlet and triplet of **COT** and the B3LYP/6-31+G(d,p) for the singlet and triplet of **mCP**, and UB3LYP/6-31+G(d,p) for the triplet excited states of **COT**, **mCP-COT** and **mCP**.

Compound	S_1-adiabatic [eV]	Singlet AEE [eV]	S_0@S_1 geometry [eV]	T_1-adiabatic [eV]	Triplet AEE [eV]	S_0@T_1 geometry [eV]
	B3LYP/6-31+G(d,p)//CIS/6-31+G(d,p)					
**COT**	−8423.710	1.232	−8423.885	−8424.300	0.642	−8424.009
**mCP-COT**	−49229.143	1.093	−49228.657	−49229.412	0.824	−49229.047
	B3LYP/6-31+G(d,p)					
**mCP**	−34415.110	3.586	−34418.171	−34415.845	2.851	−34418.355
				UB3LYP/6-31+G(d,p)		
**COT**				−8424.312	0.630	−8423.874
**mCP-COT**				−49229.508	0.729	−49229.085
**mCP**				−34415.573	3.123	−34418.310

Supplementary Table 6. Summary of photophysical parameters for BSBCz-EH neat and blend (mCP-COT) thin films.

	**PLQY** (%)	**Lifetime** (ns)	$\boldsymbol{k}_\mathbf{r}$ (s^{-1})
Neat	68	1.39	4.9×10^8
1wt% mCP-COT	71	1.56	4.6×10^8
s3wt% mCP-COT	71	1.54	4.6×10^8
5wt% mCP-COT	72	1.53	4.7×10^8
10wt% mCP-COT	68	1.43	4.8×10^8
20wt% mCP-COT	67	1.30	5.2×10^8
50wt% mCP-COT	53	1.13	4.7×10^8
90wt% mCP-COT	34	1.10 (0.41, 1.34)	3.1×10^8

Supplementary Section 4: ^{1}H (500 MHz, $CDCl_3$) and ^{13}C NMR (125 MHz, $CDCl_3$) spectra of all compounds.

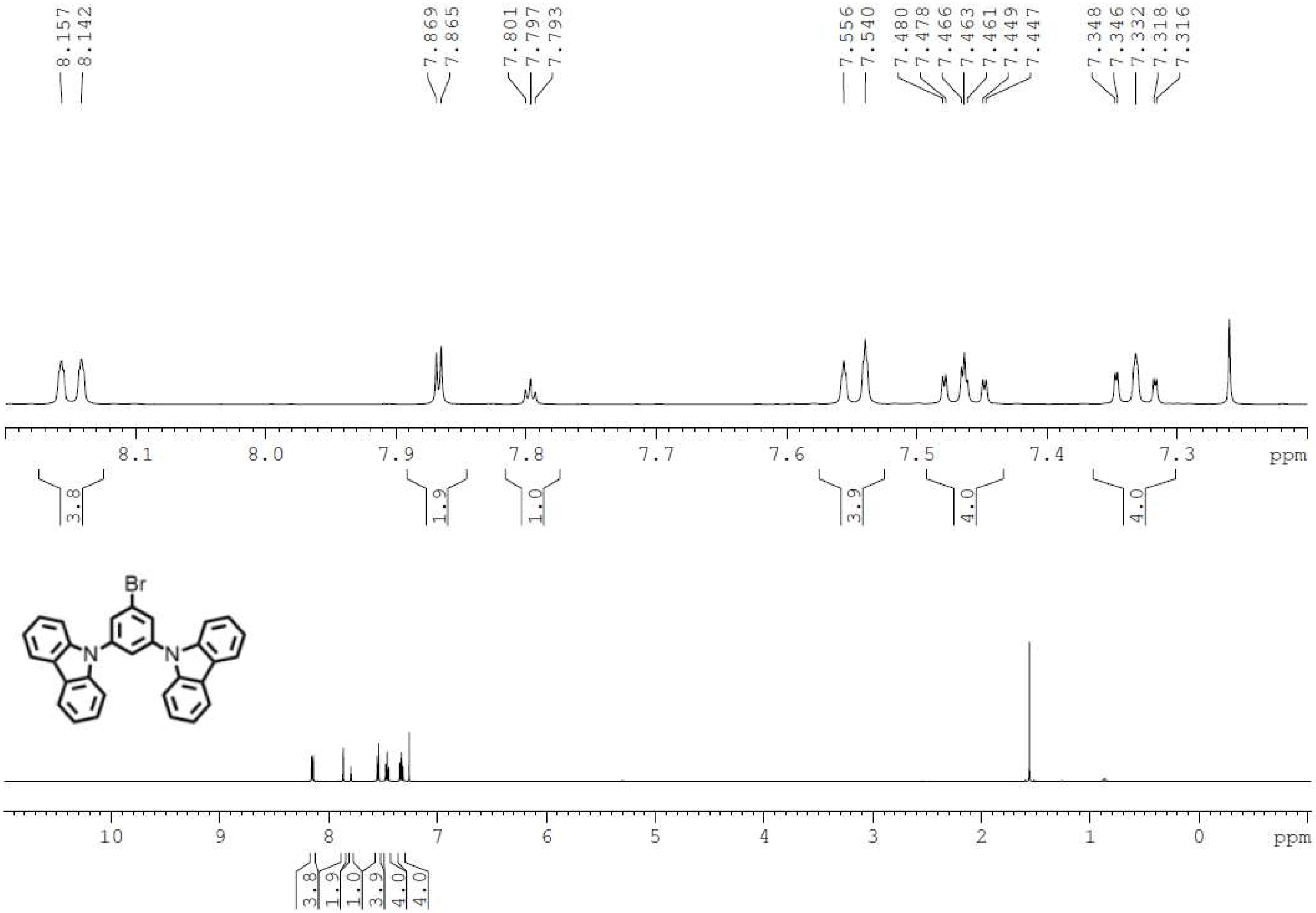

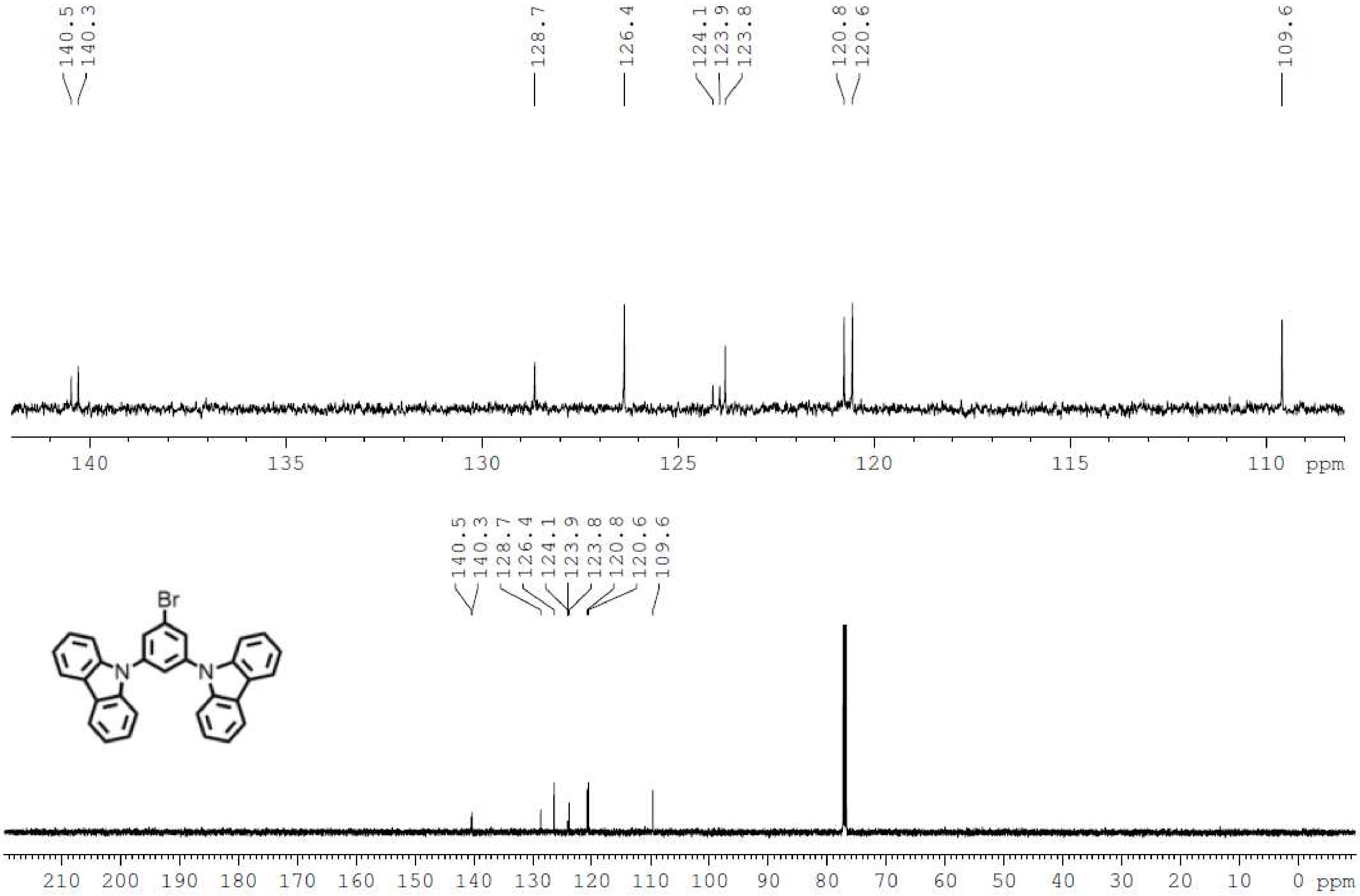
140.5
140.3
128.7
126.4
124.1
123.9
123.8
120.8
120.6
109.6
140 135 130 125 120 115 110 ppm
140.5
140.3
128.7
126.4
124.1
123.9
123.8
120.8
120.6
109.6
Br
N
N
210 200 190 180 170 160 150 140 130 120 110 100 90 80 70 60 50 40 30 20 10 0 ppm

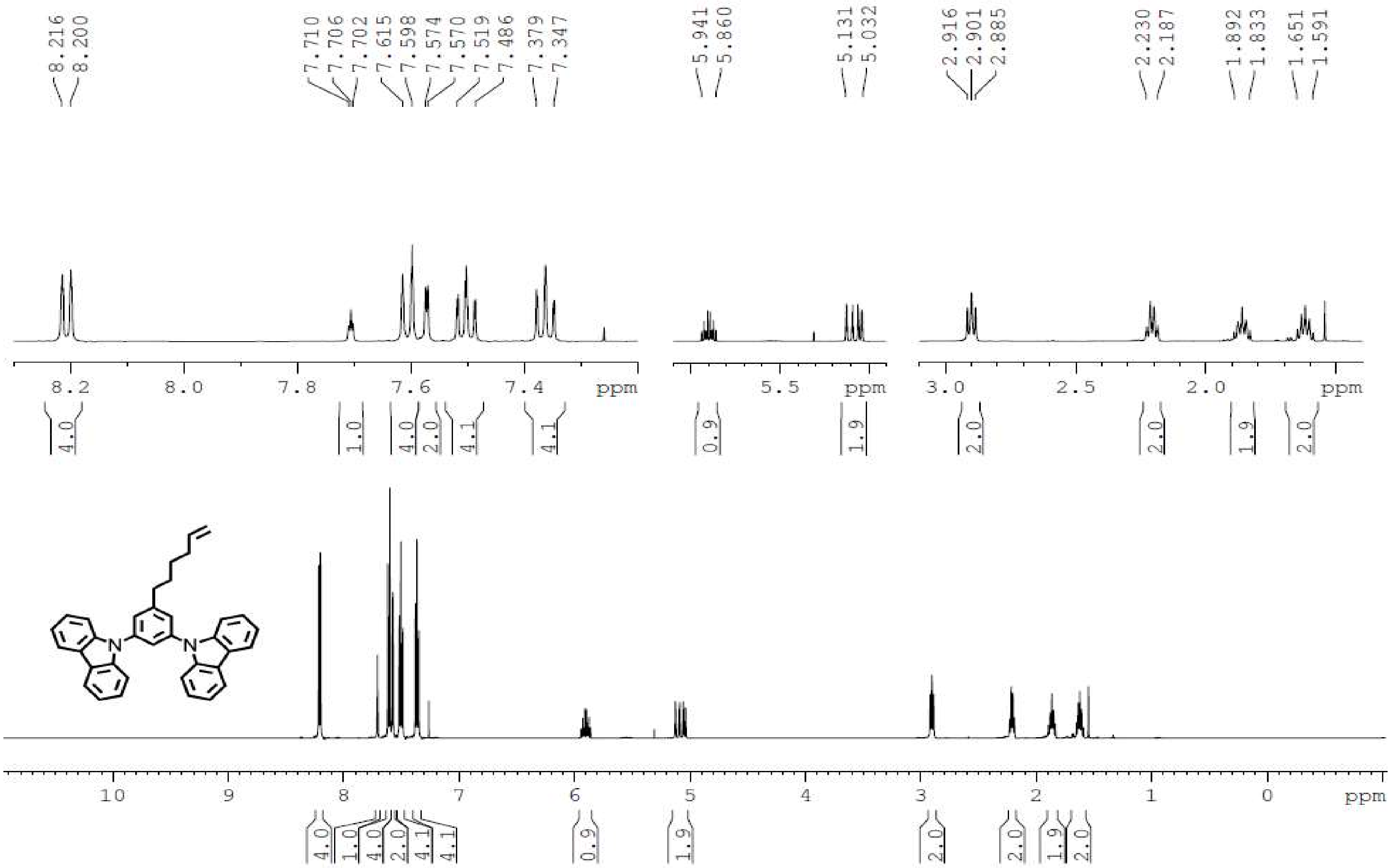

8.216
8.200
7.710
7.706
7.702
7.615
7.598
7.574
7.570
7.519
7.486
7.379
7.347
5.941
5.860
5.131
5.032
2.916
2.901
2.885
2.230
2.187
1.892
1.833
1.651
1.591
ppm
N
N

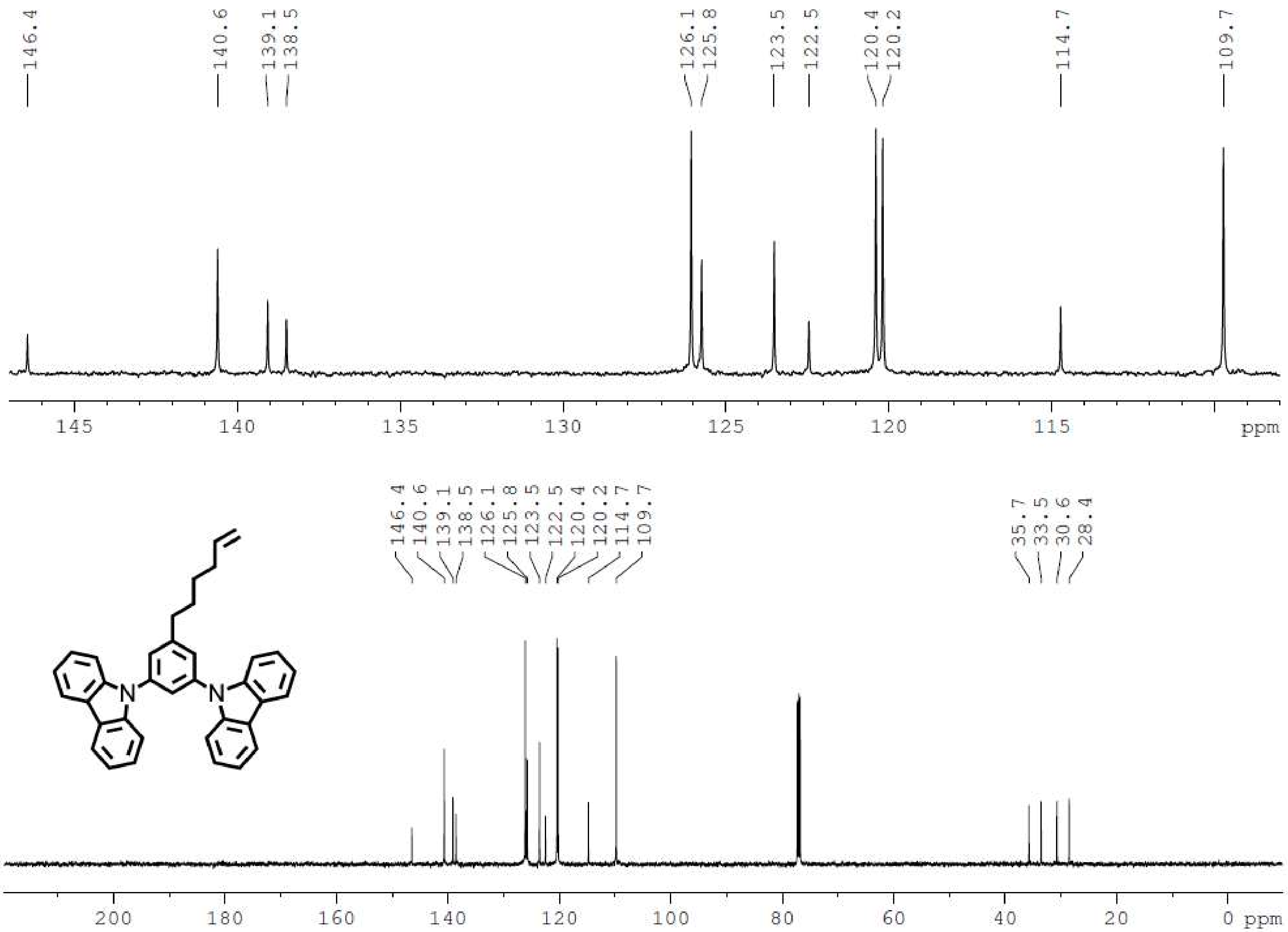
146.4
140.6
139.1
138.5
126.1
125.8
123.5
122.5
120.4
120.2
114.7
109.7
145 140 135 130 125 120 115 ppm
146.4
140.6
139.1
138.5
126.1
125.8
123.5
122.5
120.4
120.2
114.7
109.7
35.7
33.5
30.6
28.4
N
N
200 180 160 140 120 100 80 60 40 20 0 ppm

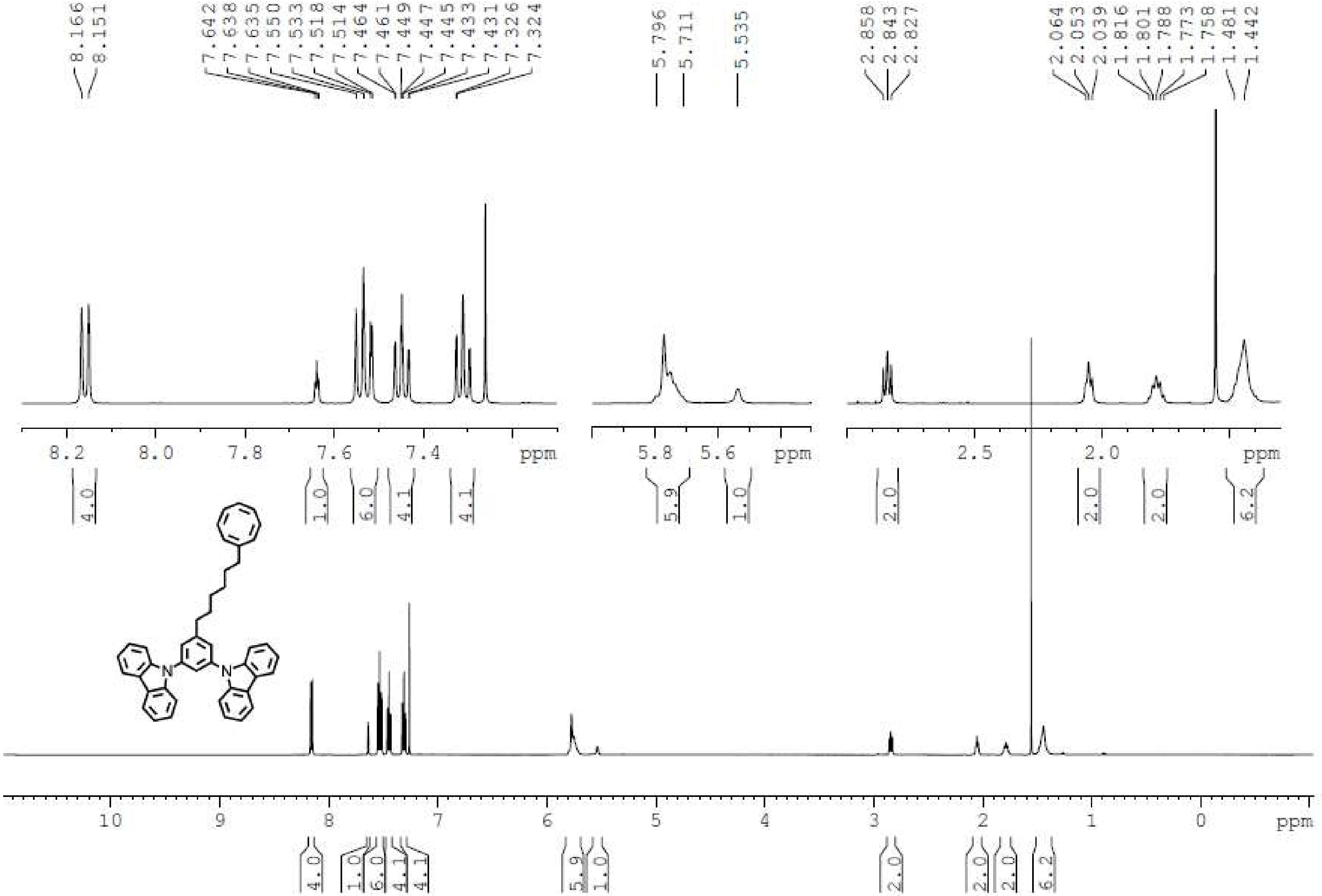
8.166
8.151
7.642
7.638
7.635
7.550
7.533
7.518
7.514
7.464
7.461
7.449
7.447
7.445
7.433
7.431
7.326
7.324
5.796
5.711
5.535
2.858
2.843
2.827
2.064
2.053
2.039
1.816
1.801
1.788
1.773
1.758
1.481
1.442
8.2 8.0 7.8 7.6 7.4 ppm
5.8 5.6 ppm
2.5 2.0 ppm
4.0 1.0 6.0 4.1 4.1
5.9 1.0
2.0 2.0 2.0 6.2
N
N
10 9 8 7 6 5 4 3 2 1 0 ppm

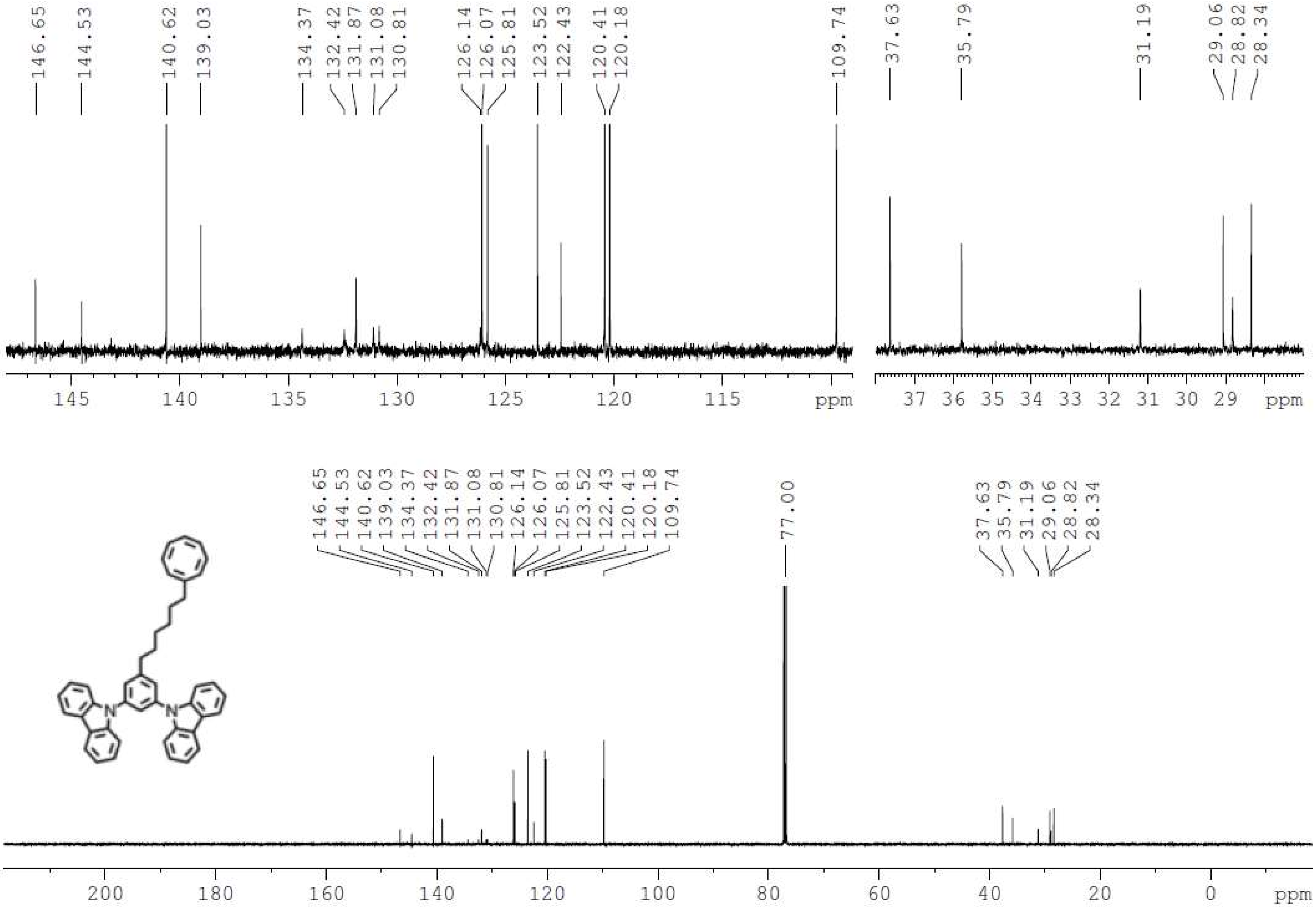
146.65
144.53
140.62
139.03
134.37
132.42
131.87
131.08
130.81
126.14
126.07
125.81
123.52
122.43
120.41
120.18
109.74
37.63
35.79
31.19
29.06
28.82
28.34
145 140 135 130 125 120 115 ppm
37 36 35 34 33 32 31 30 29 ppm
77.00
200 180 160 140 120 100 80 60 40 20 0 ppm

References:

1 Wu, C. *et al.* Versatile donor-pi-acceptor-type aggregation-enhanced emission active fluorophores as both highly efficient nondoped emitter and excellent host. *ACS Appl. Mater. Interfaces* **9**, 32946–32956 (2017).

2 Sun, D. *et al.* Oligosiloxane functionalized with pendant (1,3-bis(9-carbazolyl)benzene) (mCP) for solution-processed organic electronics. *Chemistry* **20**, 16233–16241 (2014).

3 Roger, B. A. *et al.* Cyanine fluorophore derivatives with enhanced photostability. *Nat. Methods* **9**, 68–71 (2011).

4 Lin, M.-S. *et al.* Incorporation of a CN group into mCP: a new bipolar host material for highly efficient blue and white electrophosphorescent devices. *J. Mater. Chem.* **22**, 16114–16120 (2012).

5 Lo, S.-C. *et al.* The synthesis and properties of iridium cored dendrimers with carbazole dendrons. *Org. Electronics* **7**, 85–98 (2006).

6 Speer, M. E., Sterzenbach, C. & Esser, B. Evaluation of cyclooctatetraene-based aliphatic polymers as battery materials: synthesis, electrochemical, and thermal characterization supported by DFT calculations. *ChemPlusChem* **82**, 1274–1281 (2017).

7. Schmerl, N. M., Khodakov, D. A., Stapleton, A. J., Ellis, A. V. & Andersson, G. G. Valence band structure of pdms surface and a blend with MWCNTs: A UPS and MIES study of an insulating polymer. *Appl. Surf. Sci*. **353**, 693–699 (2015).

8. Zhao, Y., Chen, J., Chen, W. & Ma, D. Poly(3,4-ethylenedioxythiophene):poly(styrenesulfonate)/MoO_3 composite layer for efficient and stable hole injection in organic semiconductors. *J. Appl. Phys.* **111**, 043716 (2012).

9. Kröger, M. *et al*. Role of the Deep-lying electronic states of MoO_3 in the enhancement of hole-injection in organic thin films. *Appl. Phys. Lett.* **95**, 123301 (2009).

10. Zhang, Y. & Forrest, S. R. Existence of continuous-wave threshold for organic semiconductor lasers. *Phys. Rev. B* **84**, 241301 (2011).
